# Diagnostic value of diffusion-weighted imaging with synthetic b-values in breast tumors: comparison with dynamic contrast-enhanced and multiparametric MRI

**DOI:** 10.1007/s00330-020-07094-z

**Published:** 2020-08-11

**Authors:** Isaac Daimiel Naranjo, Roberto Lo Gullo, Carolina Saccarelli, Sunitha B. Thakur, Almir Bitencourt, Elizabeth A. Morris, Maxine S. Jochelson, Varadan Sevilimedu, Danny F. Martinez, Katja Pinker-Domenig

**Affiliations:** 1 Department of Radiology, Breast Imaging Service, Memorial Sloan Kettering Cancer Center, 300 E 66th Street, New York, NY 10065 USA; 2 Department of Radiology, Breast Imaging Division, Istituto Europeo di Oncologia, Via Giuseppe Ripamonti, 435, 20141 Milano, Italy; 3 Department of Medical Physics, Memorial Sloan Kettering Cancer Center, 1275 York Ave, New York, NY 10065 USA; 4 Department of Imaging, A.C.Camargo Cancer Center, SP São Paulo, Brazil; 5 Department of Epidemiology and Biostatistics, Memorial Sloan Kettering Cancer Center, 1275 York Ave, New York, NY 10065 USA; 6 Department of Biomedical Imaging and Image-guided Therapy Division of Molecular and Gender Imaging, Medical University of Vienna, Waehringer Guertel 18-20, 1090 Vienna, Austria

**Keywords:** Breast tumors, Image analysis, Diagnostic imaging, Diffusion magnetic resonance imaging, Echo-planar imaging

## Abstract

**Objectives:**

To assess DWI for tumor visibility and breast cancer detection by the addition of different synthetic b-values.

**Methods:**

Eighty-four consecutive women who underwent a breast-multiparametric-MRI (mpMRI) with enhancing lesions on DCE-MRI (BI-RADS 2–5) were included in this IRB-approved retrospective study from September 2018 to March 2019. Three readers evaluated DW acquired b-800 and synthetic b-1000, b-1200, b-1500, and b-1800 s/mm^2^ images for lesion visibility and preferred b-value based on lesion conspicuity. Image quality (1–3 scores) and breast composition (BI-RADS) were also recorded. Diagnostic parameters for DWI were determined using a 1–5 malignancy score based on qualitative imaging parameters (acquired + preferred synthetic b-values) and ADC values. BI-RADS classification was used for DCE-MRI and quantitative ADC values + BI-RADS were used for mpMRI.

**Results:**

Sixty-four malignant (average = 23 mm) and 39 benign (average = 8 mm) lesions were found in 80 women. Although b-800 achieved the best image quality score, synthetic b-values 1200–1500 s/mm^2^ were preferred for lesion conspicuity, especially in dense breast. b-800 and synthetic b-1000/b-1200 s/mm^2^ values allowed the visualization of 84–90% of cancers visible with DCE-MRI performing better than b-1500/b-1800 s/mm^2^. DWI was more specific (86.3% vs 65.7%, *p* < 0.001) but less sensitive (62.8% vs 90%, *p* < 0.001) and accurate (71% vs 80.7%, *p* = 0.003) than DCE-MRI for breast cancer detection, where mpMRI was the most accurate modality accounting for less false positive cases.

**Conclusion:**

The addition of synthetic b-values enhances tumor conspicuity and could potentially improve tumor visualization particularly in dense breast. However, its supportive role for DWI breast cancer detection is still not definite.

**Key Points:**

• *The addition of synthetic b-values (1200–1500 s/mm*^*2*^*) to acquired DWI afforded a better lesion conspicuity without increasing acquisition time and was particularly useful in dense breasts.*

• *Despite the use of synthetic b-values, DWI was less sensitive and accurate than DCE-MRI for breast cancer detection.*

• *A multiparametric MRI modality still remains the best approach having the highest accuracy for breast cancer detection and thus reducing the number of unnecessary biopsies.*

**Electronic supplementary material:**

The online version of this article (10.1007/s00330-020-07094-z) contains supplementary material, which is available to authorized users.

## Introduction

Diffusion-weighted imaging (DWI) is increasingly incorporated into breast MRI protocols worldwide [1–3]. DWI using apparent diffusion coefficient (ADC) mapping has reported sensitivities of up to 96% and specificities of up to 100% for breast cancer detection [4, 5]. Currently, the prime focus of DWI is to differentiate between benign and malignant lesions to prevent unnecessary breast biopsies. With the recent concerns regarding the safety of gadolinium-based contrast agents (GBCAs) [6–8], DWI has been proposed as a promising alternative to dynamic contrast-enhanced magnetic resonance imaging (DCE-MRI) to detect early breast cancer without the costs and safety concerns associated with GBCAs [9–14].

Several studies have demonstrated that the sensitivity of unenhanced MRI with DWI was equal to or superior to mammography [4, 15]; however, there is still room for improvement [16]. Diffusion sensitivity, better known as “b-value,” has important implications for tumor conspicuity and can be controlled by modifying the magnitude and duration of the diffusion gradients. Higher b-values seem to improve lesion conspicuity by suppressing the normal breast tissue and decreasing the T2 shine-through effect [17]. Nevertheless, they require long examination times and the image quality may be compromised due to a low signal-to-noise ratio [18]. Synthetic b-values may overcome these limitations. Synthetic b-values are generated through a mathematical computation technique from at least two different lower b-values in a voxelwise manner [19–21] without increasing the scan time or reducing the image quality (in fact, synthetic b-values present a higher image quality than the acquired b-values) [22] and therefore have the potential to improve the sensitivity of breast cancer detection.

The aim of our study was to assess lesion visibility and the diagnostic performance of DWI for breast cancer detection by the addition of different synthetic b-values.

## Materials and methods

### Patients

This single-institution study and retrospective data analysis was approved by the Institutional Review Board and was conducted in compliance with the Health Insurance Portability and Accountability Act.

Between September 2018 and March 2019, 84 consecutive women who underwent a breast MRI examination (including DCE-MRI and DWI) at our institution and fulfilled the inclusion criterion of presenting with an enhancing lesion on DCE-MRI (categories 2–5 of the Breast Imaging Reporting and Data System (BI-RADS)) were included in this study. Indications for an MRI examination in these women included screening (46.2%), extent of the disease and surgical planning (33.8%), inconclusive findings in other imaging modalities (6.2%), MRI follow-up examinations for previous findings (5%), evaluation of recurrent tumor (6.3%), and nipple discharge (2.5%). Patients undergoing chemotherapy; pregnant women; and those undergoing examinations without DWI series, a biopsy-proven histology, or at least lesion stability for 24 months were excluded.

Due to technical failure of the DWI sequence and the presence of a clip/biopsy change generating obvious image distortion, four patients were excluded, resulting in a final study population of 80 women (mean age 48.1 ± 12.5 years; range 26–76 years) with 103 breast lesions. Forty-five of these patients were pre-menopausal (56.25%) and 35 were post-menopausal (43.75%).

### MRI examination

All the examinations were performed using a 3-T MRI scanner (Discovery MR750; GE Healthcare) with a dedicated 16-channel phased-array breast coil (Sentinelle Coil, Hologic). All the women underwent a state-of-the-art multiparametric MRI (mpMRI) protocol with T2-weighted imaging, DCE-MRI, and DWI. DW images were always acquired before contrast agent injection using a single-shot echo-planar imaging (EPI) sequence with 0 and 800 s/mm^2^ b-values (Supplemental Table 1, Supplemental Digital Content 1). Synthetic DWI b-values 1000, 1200, 1500, and 1800 s/mm^2^ were automatically generated from the acquired b-values using a built-in software. Synthetic b-values were selected based on previous literature [23, 24].

### Image analysis

Three dedicated breast radiologists (I.D., R.L., and C.S.) with 4–5 years of experience in interpretation of multiparametric breast MRI evaluated images independently using OsiriX v.9.0 software (OsiriX). Readers were aware of the presence of lesions in all the examinations but were blinded to any clinical information and conventional and prior imaging.

#### DWI

Readers first assessed DW images (b-800, b-1000, b-1200, b-1500, and b-1800 s/mm^2^) and ADC maps blinded to the DCE-MRI. For all the lesions, visibility using each b-value (yes/no), location, and laterality were recorded. If more than one lesion was visible, all lesions were recorded. A visual grading image quality score (1 = bad quality, 2 = average, 3 = good quality) was assigned by each reader for all the b-values based on artifacts and fat suppression. In addition, a preferred b-value was selected by each reader based on lesion conspicuity defined as the visual difference in lesion contrast with the surrounding parenchyma.

One 2D region of interest (ROI) per lesion and reader was drawn manually on ADC maps derived from acquired b-values using the OsiriX v.9.0 software (OsiriX). The ROI was placed in a slice containing the tumor maximum diameter and within the area with the lowest ADC values.

Each reader assigned a 1–5 malignancy score to DW images (from 1 = non-suspicious to 5 = highly suspicious) using acquired and preferred synthetic b-values for each visible lesion. The criteria for this score included qualitative parameters based upon the previous literature [15, 25] as well as quantitative ADC values extracted from ADC maps as shown in Table 1. Scores 4 and 5 were considered suspicious for malignancy, whereas scores 1, 2, and 3 were considered non-suspicious.

**Table 1:** Criteria for DWI malignancy score

Descriptor	DWI + ADC map	
	Suspicious	Not suspicious
Internal signal	Heterogeneous	Homogeneous
Shape	Irregular/angulated	Round/oval
ADC	≤ 1.3 × 10^−3^ mm^2^/s	> 1.3 × 10^−3^ mm^2^/s

*DWI*, diffusion-weighted image; *ADC*, apparent diffusion coefficient

#### DCE-MRI

After a wash-out period of at least 21 days, DCE-MRI alone was read. Readers classified lesions according to BI-RADS classification [26]. Lesions categorized as BI-RADS 2/3 were considered non-suspicious, whereas categories BI-RADS 4/5 were considered suspicious for malignancy.

Consequently, the results for both readings were reviewed in consensus for missed lesions on DWI or a lesion mis-match between DCE-MRI and DWI. In the case of mis-matched or missed lesions on DWI by one or two of the readers, they were asked to obtain ADC values for lesion categorization. Lesions missed by all the readers were excluded for categorization. The mean ADC values for all the lesions across readers were then determined (Supplemental Table 2, Supplemental Digital Content 1). Categories for breast composition of fibroglandular tissue (FGT) were recorded for each examination based on its report (A-almost entirely fat, B-scattered FGT, C-heterogeneous FGT, and D-extreme FGT).

#### Multiparametric MRI

mpMRI with DWI and DCE-MRI was evaluated using an ADC cutoff value of 1.3 × 10^−3^ mm^2^/s as recommended by the European Society of Breast Imaging [15]. A final lesion classification was given as follows: If a BI-RADS 4 or 5 was assigned on DCE-MRI, an ADC > 1.3 × 10^−3^ mm^2^/s was required to assign a final classification as non-suspicious. If a BI-RADS 2 or 3 was assigned, an ADC ≤ 1.3 × 10^−3^ mm^2^/s was required to assign a final classification as suspicious.

### Histopathology

The final diagnosis was established by histopathology using image-guided needle biopsy for the majority of the lesions (*n* = 98). In the event of discordant findings between histopathology and imaging, the final diagnosis was established surgically (*n* = 2). Benignity was confirmed in three lesions by imaging follow-up of up to 24 months.

### Statistical analysis

All calculations were performed using SPSS 25.0 (IBM) and SAS 9.4 (SAS Institute) in a per-lesion analysis. Median and mean ranks were calculated for image quality and preferred b-values. Sensitivity, specificity accuracy, and their 95% confidence intervals (CI) were calculated for the imaging methods and averaged over the three readers [27]. Likewise, diagnostic parameters for breast cancer detection were obtained for each imaging modality for lesions stratified by size (small lesions ≤ 10 mm and lesions > 1 mm). Receiver operating curves (ROC) were obtained using the PROC GLIMMIX statement in SAS 9.4 (SAS Institute) by treating each reader’s assessment as a fixed effect and estimating a robust (sandwich) measure of variance to account for the correlation between multiple readers [28]. The epidemiological parameters and the areas under the ROC curves (AUC) were compared between the three diagnostic modalities by using chi-square tests [29]. Bonferroni’s correction was made for multiple pairwise comparisons (*α* = 0.016).

Cohen’s *κ* and concordance correlation coefficient statistics were used to determine the concordance between imaging methods and readers [30]. The inter-reader agreement between ADC measurements was documented using Bland–Altman plots where the ADC mean was used.

## Results

One hundred and three lesions (91 enhancing masses and 12 non-mass lesions (NMLE)) from 80 women were assessed including 64 malignant tumors (mean size 23 mm; range 5–100 mm) and 39 benign lesions (mean size 8 mm; range 5–22 mm). Histological results are shown in Table 2. Breast composition of these women included 37 (46.2%) women within categories A/B and 43 (53.8%) women within categories C/D.

**Table 2:** Histopathology of all lesions stratified by benignity and malignancy

Benign lesions (*n* = 39) (37.9%)		Malignant lesions (*n* = 64) (62.1%)		
Fibroadenoma	16 (15.6%)	IDC	Total	53 (51.5%)
Fibrocystic changes	4 (3.9%)		Low grade	4 (3.9%)
Benign breast parenchyma	2 (1.9%)		Intermediate grade	25 (24.3%)
			High grade	24 (23.3%)
			DCIS associated	12 (22.6%)
ALH	1 (0.9%)	ILC		5 (4.8%)
PASH	5 (4.9%)			
Papilloma	2 (1.9%)			
ADH	1 (0.9%)	DCIS		6 (5.8%)
Hamartoma	1 (0.9%)			
Benign unchanged (at least 2-year follow-up)	3 (2.9%)			
Stromal fibrosis and adenosis	3 (2.9%)			
LCIS	1 (0.9%)			

*ALH*, atypical lobular hyperplasia; *PASH*, pseudoangiomatous hyperplasia; *ADH*, atypical ductal hyperplasia; *IDC*, invasive ductal carcinoma; *ILC*, invasive lobular carcinoma; *DCIS*, ductal carcinoma in situ; *LCIS*, lobular carcinoma in situ

### Visual grading image quality score

Acquired b-800 s/mm^2^ was the best rated by all the readers in terms of image quality, whereas synthetic b-1800 s/mm^2^ was the worst rated. Mean image quality scores for the different b-values are summarized in Supplemental Table 3 in the Electronic Supplementary Material.

### Overall lesion visibility with DWI

No mis-matched lesions with DCE-MRI were identified. The percentage of enhancing lesions identified on DWI was 82.5% for reader 1 (r1), 78.6% for reader 2 (r2), and 81.5% for reader 3 (r3). DWI visualized 84–90% of the malignant enhancing lesions. Results of missed lesions by reader are summarized in Table 3. Readers missed the same number of malignant lesions in acquired b-800 s/mm^2^ and synthetic b-1000/b-1200 s/mm^2^ images (r1: 8, r2: 10, r3: 6). Fewer tumors were visualized at higher b-values b-1500 (r1: 10, r2: 12, r3: 6) and b-1800 s/mm^2^ (r1: 11, r2: 14, r3: 11).

**Table 3:** Lesions not visible with DWI for all readers

Histology	Reader 1	Reader 2	Reader 3	Lesions missed by all readers
Benign	10	12	13	5
Malignant	8	10	6	5
Total of missed lesions	18 (17.5%)	22 (21.4%)	19 (18.5%)	10 (9.7%)
Mean lesion size (range)	7.7 mm (5–20 mm)	8.7 mm (5–22 mm)	7.9 mm (5–20 mm)	8.3 mm (5–20 mm)
% of lesions visualized with DWI	82.5%	78.6%	81.5%	

**Fig. 1:**
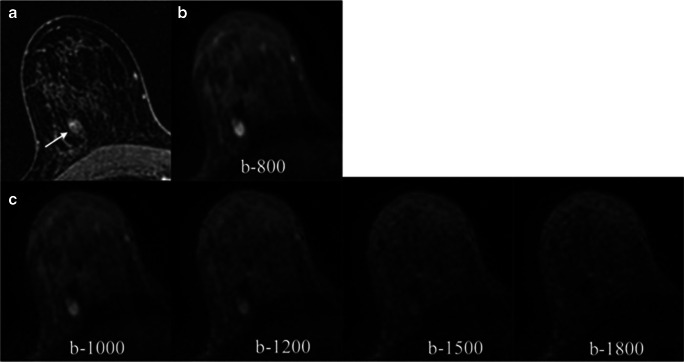
Axial contrast-enhanced (**a**), acquired DWI (**b**), and synthetic DWI (**c**) images of a patient with a 10-mm biopsy-proven fibroadenoma in the third posterior depth of the right breast. This benign lesion is less conspicuous at higher b-values being barely visible at b-1500 and b-1800 s/mm^2^. ADC mean value for this lesion was 1850 × 10^−6^ mm^2^/s. This lesion was correctly categorized as non-suspicious in both DWI and DCE-MRI. DWI, diffusion-weighted imaging; ADC, apparent diffusion coefficient; DCE-MRI, dynamic contrast-enhanced magnetic resonance imaging

DWI performed poorly for lesion visibility of small tumors (< 10 mm) and benign lesions, especially synthetic b-1800 s/mm^2^ values. Results for missed lesions stratified by reader and b-values are summarized in Supplemental Table 4 in the Electronic Supplementary Material. Figure 1 shows an example of a benign lesion not seen by extremely high b-values.

**Fig. 2:**
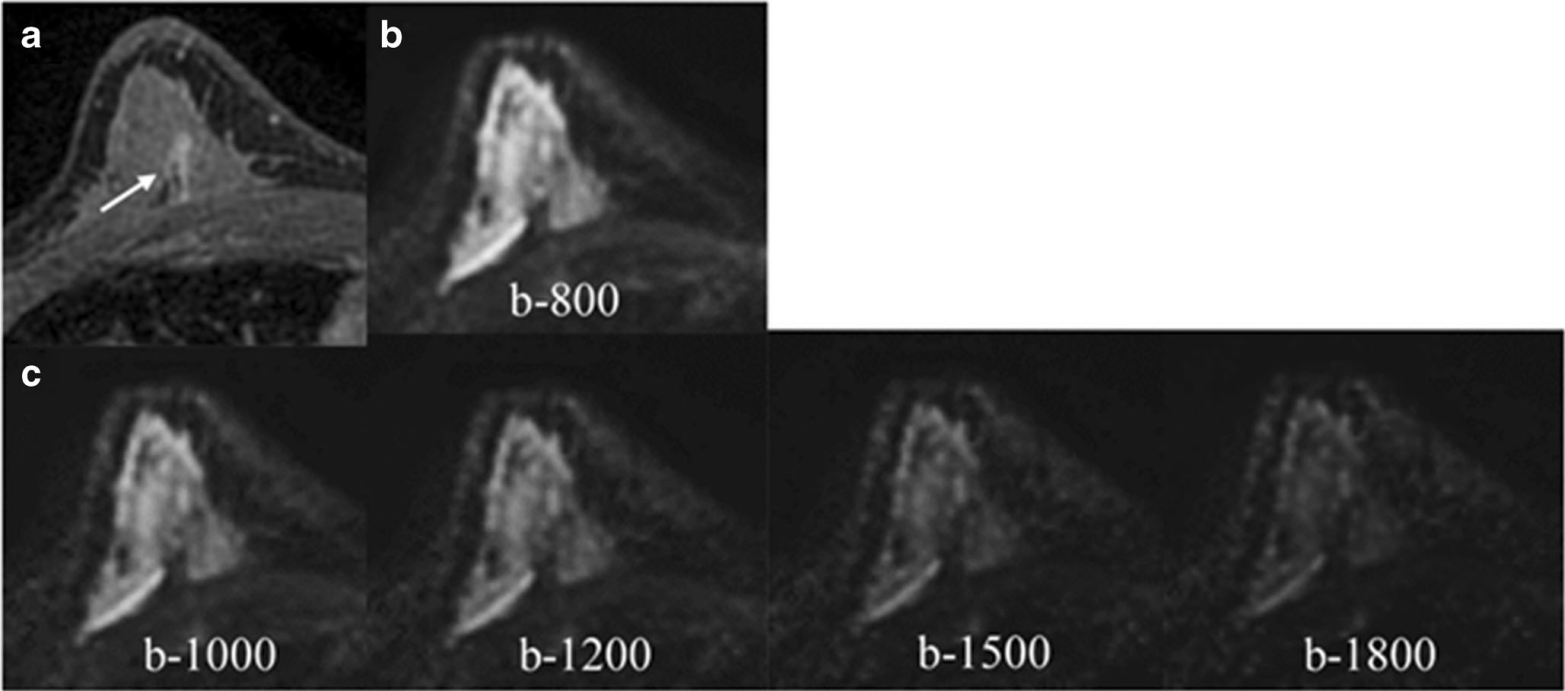
Axial images of a biopsy-proven DCIS in a patient with dense breast. Contrast-enhanced image (**a**) shows a 14-mm non-mass enhancement in the posterior third of the right breast (arrow) in the middle third. All readers missed this lesion on DWI which was indistinguishable either in the acquire DWI b-800 (**b**) or the synthetic b-values (**c**). DCIS, ductal carcinoma in situ; DWI, diffusion-weighted imaging

Among the lesions missed by all the readers (10 enhancing lesions representing 9.7%), one 8-mm malignant NMLE was not visible on DWI due to being included in the gap between slices in DWI. The remaining nine lesions included 4 NMLE (mean size 11.7 mm; range 6–20 mm), of which two were < 10 mm and 5 masses < 10 mm (mean size 5.6 mm; range 5–8 mm) with an overall mean lesion size of 8.3 mm. Histology of missed tumors was benign in five cases (one benign breast parenchyma (NMLE), one papilloma (mass), two fibroadenomas (masses), and one unchanged lesion on follow-up (mass)) and malignant in four cases (two ductal carcinomas in situ (DCIS) (NMLE), one invasive ductal carcinoma (IDC) grade I (mass), and one invasive lobular carcinoma (ILC) (NMLE)). Figure 2 shows an example of a missed lesion by all the readers.

### Preferred synthetic b-value

**Fig. 3:**
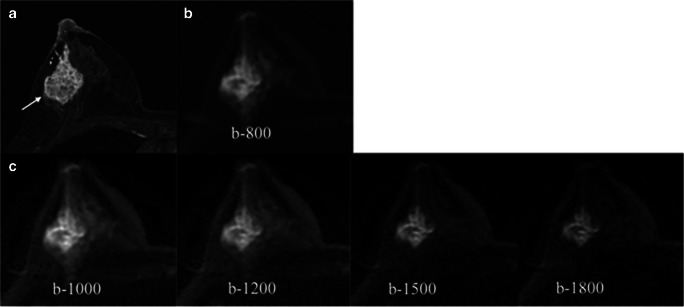
Axial images of a 47-year-old woman with dense breasts. Contrast-enhanced image (**a**) shows a 52-mm NMLE in the outer quadrants of the right breast. Biopsy proved a high-grade invasive ductal carcinoma. DWI acquired image (**b**) and synthetic images (**c**) are depicted. An increased lesion-to-normal-tissue contrast at synthetic high b-values due to suppression of the glandular signal is noticed. ADC mean value for this lesion was 1266 × 10^−6^ mm^2^/s. This lesion was correctly categorized as suspicious in both DWI and DCE-MRI. NMLE, non-mass lesion; DWI, diffusion-weighted imaging; ADC, apparent diffusion coefficient; DCE-MRI, dynamic contrast-enhanced magnetic resonance imaging

Synthetic b-values 1200–1500 s/mm^2^ provided the best lesion conspicuity across all readers. Reader 1 preferred b-1500 s/mm^2^, while readers 2 and 3 preferred b-1200 s/mm^2^. Benign lesions were more conspicuous at lower b-values, while malignant tumors appeared brighter than the surrounding parenchyma at high b-values (Fig. 3). Readers preferred lower b-values for benign lesions (r1: b-1000; r2: b-800; and r3: b-1200 s/mm^2^) and higher b-values for malignant tumors (r1: b-1500; r2: b-1200; and r3: b-1500 s/mm^2^). This was particularly relevant in relation to the amount of FGT. Twenty-nine malignant lesions were found within breast composition categories C/D. In this subgroup, readers preferred higher b-values (r1: b-1500; r2: b-1200; and r3: b-1500 s/mm^2^).

### Breast cancer detection

Diagnostic parameters for DWI, DCE-MRI, and mpMRI are summarized in Table 4.

**Table 4:** Average values of the diagnostic parameters for DWI, DCE, and multiparametric MRI

		DWI	DCE-MRI	mpMRI	*p* values		
		Average % (CI)	Average % (CI)	Average % (CI)	DWI vs DCE-MRI	DWI vs mpMRI	DCE-MRI vs mpMRI
Overall	Sensitivity	62.8% (53.1–72.5)	90% (84–96)	95.5% (91.4–99.6)	0.000	0.000	0.022
	Specificity	86.3% (79.4–93.2)	65.7% (56.2–75.2)	75.5% (66.9–84.1)	0.000	0.025	0.065
	Accuracy	71% (61.9–80.1)	80.7% (72.8–88.6)	88.2% (81.7–94.7)	0.003	0.000	0.010
Lesions ≤ 10 mm	Sensitivity	21.34% (4.6–50.8)	78.5% (49.3–95.2)	92.8% (66.2–99.8)	0.007	0.001	0.500
	Specificity	85.8% (67.3–95.9)	67.7% (47.7–84)	78.5% (58.9–91.7)	0.179	0.625	0.507
	Accuracy	64.2% (47.9–78.5)	71.5% (55.4–84.2)	83.4% (68.6–93)	0.629	0.057	0.226
Lesions > 10 mm	Sensitivity	71.7% (56.5–84)	97.9% (88.4–99.8)	97.9% (88.4–99.8)	0.000	0.000	1.000
	Specificity	83.4% (35.9–99.6)	49.9% (11.9–88.2)	83.4% (35.9–99.6)	0.500	1.000	0.625
	Accuracy	73% (58.9–84.4)	92.4% (81.4–97.9)	96.1% (86.7–99.4)	0.012	0.001	0.687

*DWI*, diffusion-weighted image; *ADC*, apparent diffusion coefficient; *DCE-MRI*, contrast-enhanced magnetic resonance image; *mpMRI*, multiparametric magnetic resonance image; *CI*, confidence interval

#### DWI and DCE-MRI

**Fig. 4:**
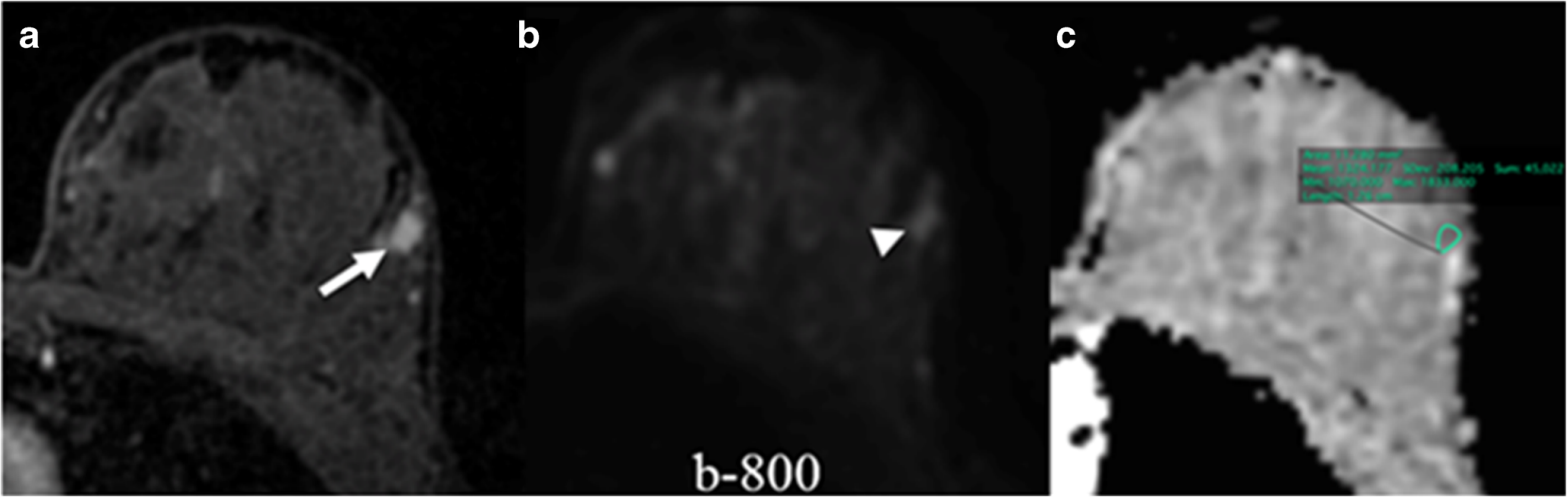
A case of a false negative in DWI. Axial images of a biopsy-proven DCIS. Contrast-enhanced image (**a**) shows a 7-mm oval irregular enhancing lesion (arrow) in the left breast. Acquired DWI b-800 (**b**) shows an oval apparently restricting lesion (arrowhead). The ADC map (**c**) shows a region of interest drawn in the corresponding location which yields an ADC mean value of 1324 × 10^−6^ mm^2^/s. This lesion was correctly categorized as malignant in DCE-MRI but categorized as benign in DWI. DWI, diffusion-weighted imaging; DCIS, ductal carcinoma in situ; ADC, apparent diffusion coefficient; DCE-MRI, dynamic contrast-enhanced magnetic resonance imaging

DWI was significantly less sensitive than DCE-MRI (62.8% vs 90%, *p* < 0.0001). However, DWI was significantly more specific than DCE-MRI (86.3% vs 65.7%, *p* < 0.0001). DWI detected fewer cancers than DCE-MRI, with 21 (reader 1) and 23 (readers 2 and 3) false negative cases on DWI (Supplemental Table 5, Electronic Supplementary Material). Figure 4 shows a case of a false negative on DWI. DWI had a significantly lower diagnostic accuracy for breast cancer detection (71% vs 80.7%, *p* = 0.003). When comparing the diagnostic performance of DWI and DCE-MRI, DWI achieved a lower AUC (0.82 vs 0.89, *p* = 0.14).

#### Multiparametric MRI

**Fig. 5:**
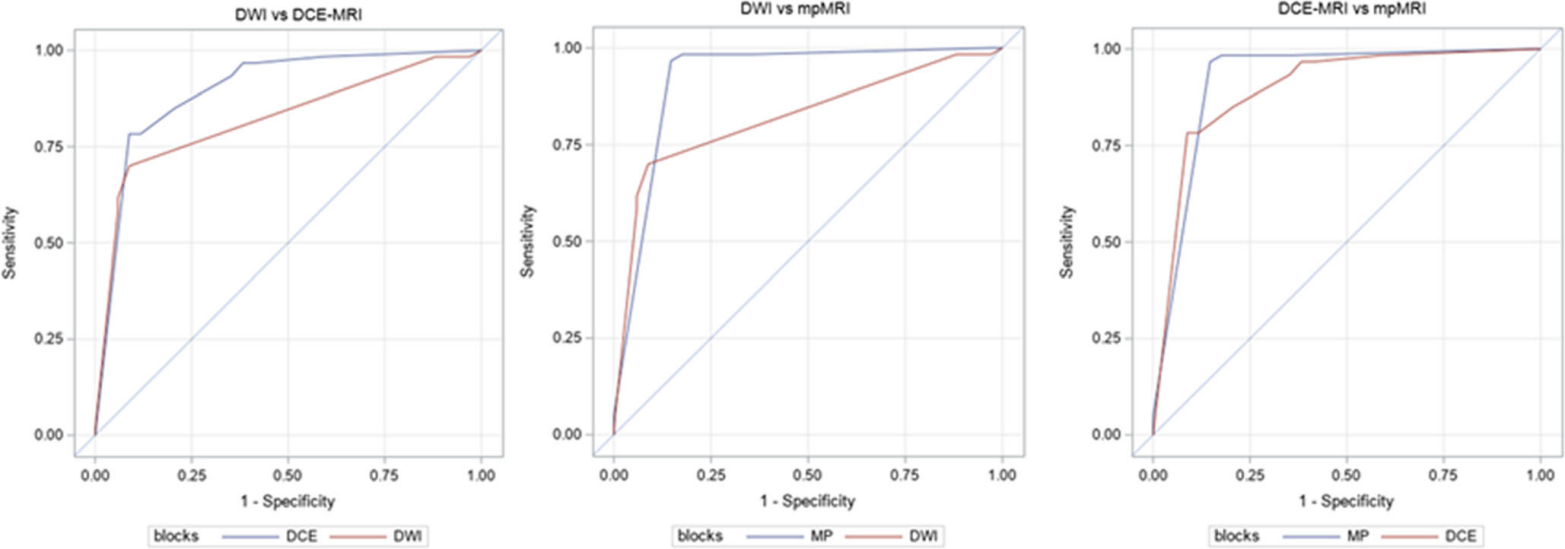
Results of pairwise comparisons between the receiver operating curves (ROCs) and the area under the curve (AUC) for each diagnostic modality

mpMRI using DCE-MRI and DWI significantly maximized the sensitivity compared with using DWI alone (95.5% vs 62.8%, *p* < 0.0001) but was not significantly different than DCE-MRI (95.5% vs 90%, *p* = 0.02). mpMRI maintained a high specificity compared with DWI alone (75.5% vs 86.3%, *p* = 0.02). mpMRI was significantly more accurate than DWI (88.2% vs 71%, *p* < 0.0001) and DCE-MRI (88.2% vs 80.7%, *p* = 0.010) across all readers. When comparing the diagnostic performance of mpMRI with DWI and DCE-MRI, mpMRI achieved a better AUC than DWI (0.92 vs 0.82, *p* = 0.05) and DCE-MRI (0.92 vs 0.89, *p* = 0.64), although these differences were not significant. Figure 5 shows ROC curves comparing DWI, DCE-MRI, and mpMRI.

When stratifying lesions by size, DWI was less accurate for lesions ≤ 10 mm than for lesions over 10 mm (64.2% vs 73%). Although mpMRI showed better accuracy than DCE-MRI in both subgroups, a significant difference was achieved only when both groups were considered. The number of false positives with mpMRI was consistently lower than with DCE-MRI in both groups, with a higher reduction in the group of lesions ≤ 10 mm. Results are shown in Supplemental Table 6 in the Electronic Supplementary Material. All misclassified benign lesions on DCE-MRI which were correctly classified by the addition of ADC values were BI-RADS 4 except two cases classified as BI-RADS 5 by reader 2 (one 13 mm fibroadenoma and one 7 mm NMLE pseudoangiomatous hyperplasia in a patient with a contralateral cancer).

### Inter-reader agreement

**Fig. 6:**
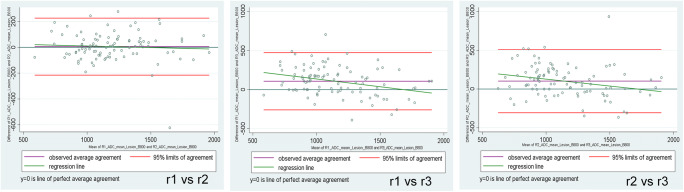
Bland–Altman plots for ADC mean inter-reader agreement. Deviation of the observed data from the line of perfect concordance (line at 45° on a square scatterplot) was used as a measure of agreement. The coefficient’s proximity to 1 indicates better agreement between two readers for that imaging parameter. ADC, apparent diffusion coefficient; r, reader

DWI inter-reader agreement was moderate to high for lesion visibility (*κ* = 0.41–0.63), preferred b-value (*κ* = 0.47–0.56), DWI malignancy score (*κ* = 0.63–0.76), and DCE-MRI assigned BI-RADS (*κ* = 0.61–0.65). Inter-reader agreement for image quality ranged from fair to high (*κ* = 0.30–0.86) across all b-values. The highest level of inter-reader agreement was for b-800 s/mm^2^ (average *κ* = 0.65), whereas the lowest *κ* value was for b-1800 s/mm^2^ (average *κ* = 0.52). Inter-reader agreement for image quality of the intermediate b-values was similar across all readers (average *κ* = 0.58). Details for inter-reader agreement are shown in Table 5. ADC achieved a high to almost perfect agreement between readers (rho_*c* = 0.90–0.73) (shown in Fig. 6).

**Table 5:** Inter-reader agreement (weighted *κ* values) for diffusion-weighted images (DWI) and dynamic contrast-enhanced MR images (DCE-MRI) parameters

			r1 vs r2	r1 vs r3	r2 vs r3	Average
DWI	b-800 s/mm^2^	Lesion visibility	0.42	0.62	0.46	0.50
		Image quality	0.86	0.60	0.48	0.65
	b-1000 s/mm^2^	Lesion visibility	0.41	0.62	0.50	0.51
		Image quality	0.76	0.52	0.46	0.58
	b-1200 s/mm^2^	Lesion visibility	0.42	0.66	0.48	0.52
		Image quality	0.76	0.52	0.46	0.58
	b-1500 s/mm^2^	Lesion visibility	0.56	0.5	0.52	0.53
		Image quality	0.76	0.52	0.46	0.58
	b-1800 s/mm^2^	Lesion visibility	0.63	0.51	0.60	0.58
		Image quality	0.79	0.46	0.30	0.52
	Preferred b-value		0.47	0.56	0.47	0.50
	Malignancy score		0.76	0.63	0.74	0.71
	*ADC mean		0.90	0.77	0.73	0.80
DCE-MRI	BI-RADS		0.65	0.63	0.61	0.63

*Apparent diffusion coefficient (ADC) values for reader’s agreement are expressed using the rho concordant correlation coefficient

## Discussion

DWI has been proposed as an unenhanced option for breast cancer screening by MRI. Synthetic b-values may improve lesion visibility without increasing the acquisition time while avoiding the disadvantages of performing DWI at very high b-values (i.e., eddy current distortions). In this study, we assessed DWI for tumor visibility and breast cancer detection by a combination of acquired b-values (800 s/mm^2^), its ADC maps, and different synthetic b-values (1000, 1200, 1500, and 1800 s/mm^2^). Acquired b-800 and synthetic b-1000/1200 s/mm^2^ values allowed the visualization of 84–90% of the malignant enhancing tumors in this study. Image quality score was lower for b-1500/1800 s/mm^2^ values; these values (especially the latter value) missed a higher number of lesions. Synthetic b-values of 1200–1500 s/mm^2^ provided the best lesion conspicuity. Benign lesions were more conspicuous at lower b-values, while malignant tumors appeared brighter than the surrounding parenchyma at higher b-values, especially in breast composition categories C/D. Despite the use of synthetic b-values, DWI was less sensitive and accurate than DCE-MRI for breast cancer detection. mpMRI maintained an excellent sensitivity and a high specificity compared with DCE-MRI and DWI alone and, therefore, significantly increased the accuracy compared with both modalities separately.

In this study, we showed that 78–82% of the lesions visible with DCE-MRI were visualized with DWI alone including both benign and malignant breast lesions. Among those lesions missed, the majority of them were either small (≤ 10 mm) or NMLE. This is in agreement with prior studies investigating DWI with ADC mapping that showed that lesions missed with DWI were either small or NMLE [16, 31, 32]. This is potentially problematic if one of the future roles of DWI is to be a reliable tool in breast cancer detection and not only in the characterization of lesions found in other imaging modalities. An improvement in the resolution of DWI sequence would be desirable to enhance cancer detection. Regarding synthetic b-values, readers were able to identify the same number of cancers using synthetic b-values of 1000/1200 s/mm^2^ and the acquired b-value of 800 s/mm^2^. In contrast, synthetic b-values of 1500 and 1800 s/mm^2^ missed more lesions, probably due to a reduction in image quality.

Most of the studies have almost exclusively focused on the visibility of breast cancer [20, 22, 33–36]; therefore, there is limited information on the conspicuity of benign breast lesions at high or synthetic b-values. While Chen et al [37] found no significant differences in conspicuity grades using b-values of 600, 800, and 1000 s/mm^2^, our results point to a difference in conspicuity. Benign lesions were more conspicuous at lower b-values, while malignant tumors appeared brighter than the surrounding parenchyma at high b-values. The increased conspicuity of breast cancer at high b-values has been demonstrated by other studies with a wider range of b-values than Chen et al [17, 38].

An improved conspicuity of malignant tumors at high b-values could be particularly helpful in dense breasts, where lesions can be mammographically masked by the large amount of FGT. In addition, an improvement of tumor visibility without contrast injection could improve the cost-effectiveness of MRI [39]. However, extremely high b-values, i.e., b-1800 s/mm^2^, have a low signal which can cause lesions located on the fat tissue to be overlooked, especially if fat is poorly suppressed [33]. In light of our results, b-1200 s/mm^2^ could be the best option for an optimal lesion visualization with the best conspicuity, which could enhance lesion characterization by a better correlation on ADC maps and more accurate ADC values.

Nevertheless, it is worth mentioning that ADC (maps and values) can only be derived from acquired DW images. Synthetic high b-value images are obtained by extrapolating signals acquired at lower b-values (e.g., 0 and 800 s/mm^2^), assuming a Gaussian model. However, diffusion in tissues is not Gaussian [2]. The calculation of synthetic high b-values is just a strategy to enhance contrast already present in lower b-value images and is potentially useful to detect and depict lesions but lacks the power of non-Gaussian diffusion to characterize tissues [40].

Although synthetic b-values over 1000 s/mm^2^ have demonstrated an improvement in tumor visualization and image quality [19, 20, 22, 34, 35, 40–43], DCE-MRI outperforms DWI for breast cancer visualization and detection with a higher sensitivity across all readers. This is in accordance with the current literature: DCE-MRI outperforms unenhanced MRI with or without supportive sequences for cancer visualization [9, 16]. In particular, tumors such as DCIS or NMLE exhibit a lower signal intensity in DWI and, therefore, are prone to be overlooked with unenhanced MRI, especially at high b-values [44]. These limitations are to be addressed to enable unenhanced MRI in a screening setting, where tumors tend to be smaller and NMLE lesions are clinically undetectable. In addition, these types of lesions account for false negative cases in DWI. In our study, a high number of IDC cases exhibited associated DCIS which could explain a slightly lower sensitivity for DWI compared with other studies [4]. Based on our results, DWI alone would currently have no role in the work-up of indeterminate lesions (e.g., BI-RADS IVa and IVb lesions), especially in small ones where its accuracy was lower mainly at the expense of a decrease in sensitivity. In this subgroup, the sensitivity and accuracy for DCE-MRI were also reduced since there is a difficulty in distinguishing morphological features. In these cases, mpMRI continued showing the best accuracy although no significant differences with DCE-MRI were found. Nevertheless, there was an additional value in the combination of DWI and DCE-MRI: a decrease in the number of false positives. This was particularly relevant in the group of small lesions ≤ 10 mm which included most of the benign lesions in our study sample. This is important to prevent unnecessary follow-up examinations in indeterminate lesions as well as benign breast biopsies, which increase costs and patient anxiety.

These results match previous publications investigating a combined DWI and DCE-MRI approach for breast cancer detection [16, 45–47].

Overall, inter-reader agreement was moderate to high for all the parameters assessed. Lesion visibility at b-800 s/mm^2^ achieved the lowest agreement, which could point to a more consistent performance of synthetic b-values for lesion visibility. Inter-reader agreement was moderate for b-values rendering the best tumor conspicuity (1200–1500 s/mm^2^). This can be explained by the fact that readers preferred a range of b-values rather than a specific value. Images at b-1800 s/mm^2^ were rated worst by all readers with respect to both lesion conspicuity and image quality.

We acknowledge several limitations in our study. Firstly, no comparison was done with acquired high b-values to maintain clinical acquisition times. Secondly, the larger size of malignant lesions compared with the benign ones and the small number of pure DCIS, ILC, and NMLE compared with invasive carcinomas presenting with a mass may affect the results and their generalization. Nevertheless, this population reflected the clinical practice in our screening and tertiary assessment center under the established inclusion criteria. Thirdly, synthetic b-values generated from different DWI sequences may yield different visual and image quality results. In our study, a single-shot EPI DWI with a short TI inversion-recovery (STIR) fat suppression sequence was used, and therefore, our results may not be extrapolated to other sequences.

In conclusion, the addition of synthetic high b-values (e.g., 1200s/mm^2^) improves tumor conspicuity without increasing the time of scan, which is particularly helpful in dense breasts. Nevertheless, the role of DWI for the visualization of NMLE and small lesions and its performance in breast cancer detection are still not definite. mpMRI remains the best modality for lesion detection with the best accuracy which is particularly helpful in MRI screening patients and obviates unnecessary biopsies in benign lesions.

## Electronic supplementary material

ESM 1 (DOCX 25 kb)

## Abbreviations

ADC: Apparent diffusion coefficient
AUC: Area under the ROC curve
CI: Confidence interval
DCIS: Ductal carcinomas in situ
DWI: Diffusion-weighted imaging
EPI: Echo-planar imaging
FGT: Fibroglandular tissue
IDC: Invasive ductal carcinoma
ILC: Invasive lobular carcinoma
NME: Non-mass enhancing
NPV: Negative predictive value
PPV: Positive predictive value
ROC: Receiver operating curve
ROI: Region of interest
STIR: Short TI inversion-recovery
