# Supplementary material for: Diagnostic value of diffusion-weighted imaging with synthetic b-values in breast tumors: comparison with dynamic contrast-enhanced and multiparametric MRI
Source: Eur Radiol. 2020 Aug 11;31(1):356–67. doi: 10.1007/s00330-020-07094-z (PMC7755636; doi:10.1007/s00330-020-07094-z)
Supplement: Supplementary file 1 — (DOCX 25 kb) [file 330_2020_7094_MOESM1_ESM.docx]

**Supplementary Digital Content**

**Supplemental Table 1.** 3T breast MRI imaging protocol

| **Sequences** | **1** | **2** | **3** | **4** | **5** | **6** |
| --- | --- | --- | --- | --- | --- | --- |
| Series Descriptor | LOC | Calibration | Axial T2 FSE | Axial DWI | Axial T1 | 3D DISCO |
| Plane |  |  | Axial | Axial | Axial | AXIAL |
| Phase FOV |  |  | 1 | 0.95 | 1 | 1 |
| Field of View (cm) | 48 | 48 | 30–36 | 30–36 | 30–36 | 30–36 |
| Slice Thickness (mm) | 12 | 10 | 3 | 3.9 | 1 | 1.1 |
| Gap (mm) |  |  | 3 | 3.9 | 1 | 1.1 |
| Saturation |  |  |  | Fat Special |  |  |
| TE1 / TE2 | 80 |  | 100 | Minimum |  | Minimum |
| TR | Min |  | 2500–6000 | 2000–17000 |  |  |
| TI TIME (Prep) |  |  |  | 210 |  |  |
| Flip Angle |  |  | 111 | 90 | 10 | 12 |
| Bandwidth (kHz) | 83kHZ | 62.5 | 83 | 250 | 62.5 | 166.67 |
| NEX |  |  | 1 | 1 (b = 0); 4 (b = 800) |  |  |
| Direction Dir. |  |  |  | ALL |  |  |
| # of Directions |  |  |  | 3 |  |  |
| b-values |  |  |  | 0, 800 |  |  |
| Matrix size | 256x256 |  | 512x512 | 256x256 | 512x512 | 512x512 |
| Frequency Direction |  | A/P | A/P | A/P | A/P | A/P |

Abbreviations: FSE: Fast Spin Echo; DISCO: Differential subsampling with cartesian ordering; A/P: Anterior-posterior; NEX: number of averages

**Supplemental Table 2.** Average of ADC mean values for each reader in benign and malignant lesions.

|  | **Reader 1** | **Reader 2** | **Reader 3** |
| --- | --- | --- | --- |
| Benign lesions | 1.45±0.25 | 1.47±0.27 | 1.40±0.29 |
| Malignant lesions | 1.05±0.16 | 1.08±0.17 | 0.91±0.20 |

*Units: × 10-3 mm2/s for all the values

**Supplemental Table 3.** DW image quality score mean values for all the readers across the b-values

| **b-value** | **Reader 1** | **Reader 2** | **Reader 3** | **Mean** |
| --- | --- | --- | --- | --- |
| 800 | 2.13 ± 0.69 | 2.15 ± 0.69 | 2.27 ± 0.5 | 2.18 ± 0.07 |
| 1000 | 2.12 ± 0.69 | 2.13 ± 0.69 | 2.26 ± 0.5 | 2.17 ± 0.07 |
| 1200 | 2.12 ± 0.69 | 2.13 ± 0.68 | 2.26 ± 0.5 | 2.17 ± 0.07 |
| 1500 | 2.12 ± 0.69 | 2.11 ± 0.67 | 2.26 ± 0.5 | 2.16 ± 0.08 |
| 1800 | 2.10 ± 0.69 | 2.03 ± 0.64 | 2.25 ± 0.49 | 2.13 ± 0.11 |

Visual grading image quality score (1– bad quality, 2– average, 3– good quality)

**Supplemental Table 4.** Lesions missed with DWI across all readers and different b-values

| **Reader** | **b-value** | **Benign** | **Malignant** | **Total of lesions** | |
| --- | --- | --- | --- | --- | --- |
| 1 | b-800 | 10 (7.6 mm; 5–20 mm) | 8 (7.8 mm; 5–14 mm) | 18 |  |
|  | b-1000 | 10 (7.6 mm; 5–20 mm) | 8 (7.8 mm; 5–14 mm) | 18 |  |
|  | b-1200 | 10 (7.6 mm; 5–20 mm) | 8 (7.8 mm; 5–14 mm) | 18 |  |
|  | b-1500 | 13 (7.4 mm; 5–20 mm) | 10 (7.6 mm; 5–14 mm) | 23 |  |
|  | b-1800 | 17 (7.3 mm; 5–20 mm) | 11 (8.2 mm; 5–14 mm) | 28 |  |
| 2 | b-800 | 12 (7.4 mm; 5–20 mm) | 10 (10 mm; 5–22mm) | 22 |  |
|  | b-1000 | 13 (7.3 mm; 5–20 mm) | 10 (10 mm; 5–22mm) | 23 |  |
|  | b-1200 | 14 (7.1 mm; 5–20 mm) | 10 (10 mm; 5–22 mm) | 24 |  |
|  | b-1500 | 18 (7.3 mm; 5–20 mm) | 12 (9.8 mm; 5–22 mm) | 30 |  |
|  | b-1800 | 27 (7.9 mm; 5–20 mm) | 14 (12.5 mm; 5–50 mm) | 41 |  |
| 3 | b-800 | 13 (7.8 mm; 5–22 mm) | 6 (8 mm; 5–14 mm) | 19 |  |
|  | b-1000 | 13 (7.8 mm; 5–22 mm) | 6 (8 mm; 5–14 mm) | 19 |  |
|  | b-1200 | 13 (7.8 mm; 5–22 mm) | 6 (8 mm; 5–14 mm) | 19 |  |
|  | b-1500 | 19 (8.6 mm; 5–20 mm) | 6 (8 mm; 5–14 mm) | 25 |  |
|  | b-1800 | 27 (8.7 mm; 5–22 mm) | 11 (12.9 mm; 5–50 mm) | 38 |  |

**Supplemental Table 5.** False negative cases with DWI

| **Reader** | **Histology** | **Tumor type** | **Mean size mm (range)** | **Total of false negatives** |
| --- | --- | --- | --- | --- |
| 1 | 13 IDC  2 IDC+DCIS  3 DCIS  3 ILC | 13 masses  1 NMLE/1mass  1 NMLE/2 masses  1 NMLE/2 masses | 14 (6–31)  30.5 (11–50)  8.3 (7–10)  11 (8–16) | 21 |
| 2 | 10 IDC  5 IDC+DCIS  4 DCIS  4 ILC | 10 masses  2 NMLE/3masses  2 NMLE/2 masses  1 NMLE/3 masses | 14.4 (6–31)  21.8 (11–50)  11.5 (7–21)  11 (8–16) | 23 |
| 3 | 10 IDC  6 IDC+DCIS  3 DCIS  4 ILC | 10 masses  2 NMLE/4masses  2 NMLE/1 mass  1 NMLE/3 masses | 12.9 (6–28)  21 (11–50)  12.6 (7–21)  11 (8–16) | 23 |

**Supplemental Table 6.** Number of false positive cases stratified by lesion size

| **Lesions ≤ 10 mm** | | | | | | **Lesions > 10 mm** | | | | | |
| --- | --- | --- | --- | --- | --- | --- | --- | --- | --- | --- | --- |
| DCE-MRI | | | mpMRI | | | DCE-MRI | | | mpMRI | | |
| r1 | r2 | r3 | r1 | r2 | r3 | r1 | r2 | r3 | r1 | r2 | r3 |
| 8 | 14 | 6 | 6 | 11 | 5 | 2 | 3 | 2 | 1 | 1 | 1 |
